# Supplementary material for: Patterns of Intron Gain and Loss in Fungi
Source: PLoS Biol. 2004 Nov 30;2(12):e422. doi: 10.1371/journal.pbio.0020422 (PMC532390; doi:10.1371/journal.pbio.0020422)
Supplement: Table S1 — Also available at http://genes.mit.edu/NielsenEtAl/. (4.3 MB ZIP). [file pbio.0020422.st001.zip › NielsenEtAl/html/397.html]

AN4782.1.NCU00600.1.MG10323.1.FG00170.1


```
 CLUSTAL W (1.82) Multiple Sequence Alignments - Introns Inserted


Sequence 1: NCU00600.1	207 aa
Sequence 2: FG00170.1	208 aa
Sequence 3: MG10323.1	210 aa
Sequence 4: AN4782.1	191 aa
Alignment Length: 213 aa
Number Identitical Residues: 125 aa
Alignment Score (without introns) 5739


MG10323.1 	MPLSLCGGSKTVQRKLVLL2GDGACGKTSLLNVFTRG2YFPTVYEPTVFE~NYVHD1IFV
NCU00600.1	MP-CGLGGSKTVQRKLVLL2GDGACGKTSLLNVFTRG2YFPTVYEPTVFE~NYVHD1IFV
FG00170.1 	MP-ALCGGSKTVQRKLVLL~GDGACGKTSLLNVFTRG2YFPTVYEPTVFE~NYVHD1IFV
AN4782.1  	MD--FCGRQKVVRRKMVLL2GDGACGKTSALNVFTRG2------EPTVFE1NYVHD1IFV
          	*     * .*.*:**:*** ********* *******       ****** ***** ***

MG10323.1 	DNVHIELSLWDTAGQEEFDRLRSLSYD1DTDLIMLCYS~VDSKDSLENVESKWVGEIADN
NCU00600.1	DNVHIELSLWDTAGQEEFDRLRSLSYD1DTDLIMLCYS~VDSKDSLENVESKWQGEIAEN
FG00170.1 	DNVHIELSLWDTAGQEEFDRLRSLSYD1DTDLIMLCYS~VDSKDSLENVESKWVGEIADN
AN4782.1  	DNVHMELSLWDTAGQEEFDRLRALSYE~DTHVIMLCFS0VDSPDSFENVASKWIEEISEN
          	****:*****************:***: **.:****:* *** **:*** ***  **::*

MG10323.1 	CPGVKLVLVALKCDLRQEQADDAAENGNATDPSQQREKKPMITYDTGLEVARRINALRYL
NCU00600.1	CPGVKIVLVALKCDLR-EATEDEEDGGANEDGAQ-REKKPMISYDQGLEVARRIKALRYL
FG00170.1 	CPGTKLVLVALKCDLR-EQGEDDENAAAGGESEQPREKKPMINYDQGLEVARRIGAQRYL
AN4782.1  	VPGVKLVLTALKCDLR----KDEYMN----------DNPNVITYEQGLAKAKEIGAVKYL
          	 **.*:**.*******    .*              ::  :*.*: **  *:.* * :**

MG10323.1 	E~CSAMRNRGVNEAFTEAARVALSVKKD---REESKCTVM
NCU00600.1	E~CSAMRNRGVNEAFTEAARVALSVKKD---REESKCVVM
FG00170.1 	E~CSAMKNRGVNEAFTEAARVALSVKKD---REEGKCIVM
AN4782.1  	E1CSAVQNRGIREAFYEAAKVALEVKAQNTGSSQSRCVIQ
          	* ***::***:.*** ***:***.** :.:. .:.:* :
```
